# Lateral line system diversification during the early stages of ecological speciation in cichlid fish

**DOI:** 10.1186/s12862-024-02214-5

**Published:** 2024-02-20

**Authors:** Duncan E. Edgley, Madeleine Carruthers, Nestory P. Gabagambi, Andrew D. Saxon, Alan M. Smith, Domino A. Joyce, Grégoire Vernaz, M. Emília Santos, George F. Turner, Martin J. Genner

**Affiliations:** 1 School of Biological Sciences, University of Bristol, Bristol, UK; 2 School of Biological and Behavioural Sciences, Queen Mary University of London, London, UK; 3 Tanzania Fisheries Research Institute, Kyela Centre, P.O. Box 98, Kyela, Mbeya Tanzania; 4 School of Natural Sciences, University of Hull, Hull, UK; 5 Department of Genetics, University of Cambridge, Downing Street, Cambridge, UK; 6 Wellcome/Cancer Research UK, Gurdon Institute, University of Cambridge, Cambridge, UK; 7 Department of Zoology, University of Cambridge, Cambridge, UK; 8 School of Natural Sciences, Bangor University, Bangor, UK

**Keywords:** Haplochromine cichlids, Evolutionary biology, Morphometrics, CT scanning, Disruptive selection, Sensory ecology

## Abstract

**Background:**

The mechanosensory lateral line system is an important sensory modality in fishes, informing multiple behaviours related to survival including finding food and navigating in dark environments. Given its ecological importance, we may expect lateral line morphology to be under disruptive selection early in the ecological speciation process. Here we quantify the lateral line system morphology of two ecomorphs of the cichlid fish *Astatotilapia calliptera* in crater Lake Masoko that have diverged from common ancestry within the past 1,000 years.

**Results:**

Based on geometric morphometric analyses of CT scans, we show that the zooplanktivorous benthic ecomorph that dominates the deeper waters of the lake has large cranial lateral line canal pores, relative to those of the nearshore invertebrate-feeding littoral ecomorph found in the shallower waters. In contrast, fluorescence imaging revealed no evidence for divergence between ecomorphs in the number of either superficial or canal neuromasts. We illustrate the magnitude of the variation we observe in Lake Masoko *A. calliptera* in the context of the neighbouring Lake Malawi mega-radiation that comprises over 700 species.

**Conclusions:**

These results provide the first evidence of divergence in this often-overlooked sensory modality in the early stages of ecological speciation, suggesting that it may have a role in the broader adaptive radiation process.

**Supplementary Information:**

The online version contains supplementary material available at 10.1186/s12862-024-02214-5.

## Background

The proliferation of forms characterising adaptive radiations is often accompanied by modifications to sensory systems [1–4], and there is considerable evidence for selection on sensory systems within the context of rapid speciation events [5]. In principle, during ecological speciation, selection will tune sensory systems to the specific requirements of organisms’ respective niches, affecting both function and morphology [6]. Thus, to investigate the speciation process we require an understanding of how organisms’ sensory systems adapt to maximise fitness within their local environments [2].

Aquatic environments can be visually and hydrodynamically noisy, so fish rely on their multiple integrated and adaptable sensory modalities to survive [7–10]. Research on the evolution of sensory systems in fish has largely focused on vision, and this has clearly demonstrated that visual adaptation is important for both initial divergence and the maintenance of reproductive isolation in sympatry [1, 2, 4]. However, the ability of fishes to survive and adapt to their environment is also dependent on the detection of water flow through the mechanoreceptive lateral line system [9, 11, 12]. Found in all fishes, this sensory modality is used for location and identification of prey, predators, and conspecifics [13–19], as well as detecting substrate proximity and mediating rheotactic responses to habitat hydrodynamics [20–22]. The primary sensory organ of the lateral line system is the neuromast, of which there are two types, forming semi-distinct modalities [7]. Superficial neuromasts are present on the surface of fish and project into the surrounding medium. In contrast canal neuromasts are recessed in fluid-filled canals within bone and soft tissue, with pores in the skin opening them to the surrounding water [7, 9, 11, 12].

Due to the range of functions of the lateral line system, we may expect selection to act on it across multiple axes of niche divergence during the early stages of an adaptive radiation [19]. For instance, if populations begin to diverge first through changes in microhabitat use [23], they are likely to require different mechanoreceptive capabilities to adapt to the occupied depths, levels of turbidity and water flow in their new environments [9]. Likewise, if speciation is initially characterised by segregation into trophic niches, we may expect an accompanying disparity in lateral line morphology [24]. In Lake Malawi cichlids, trophic resources range from highly motile fish, to cryptic infaunal invertebrates, and sessile algae [25–27], and each food is associated with specific lateral line structures in the species that consume them [15, 19, 28]. This indicates that lateral line system diversity is likely to play a role in the radiation of the cichlids of Lake Malawi.

Morphological diversification of the lateral line system has been studied across several fish clades, including research on how structure relates to function [22, 29, 30]. Variation in the morphology of the lateral line system has also been described across the major clades of haplochromine cichlids in Lake Malawi, suggesting that it may have a major role in adaptive radiation [19]. For example, within the Lake Malawi cichlid *Aulonocara stuartgranti,* an expanded canal lateral line system has been shown to facilitate feeding in the dark, implying that it is at least partially a dietary adaptation [15, 16, 19]. In other cichlids, the lateral line system has been shown to mediate interactions between males during aggressive territorial interactions [17]. However, although this morphological and functional diversity in cichlids implies an important role in adaptive diversification, there is little evidence of adaptive lateral line system divergence in a microevolutionary context. Specifically, the ecological and behavioural correlates of lateral line variation are not fully resolved, and the tempo of sensory system diversification during the speciation process is not known.

**Fig. 1:**
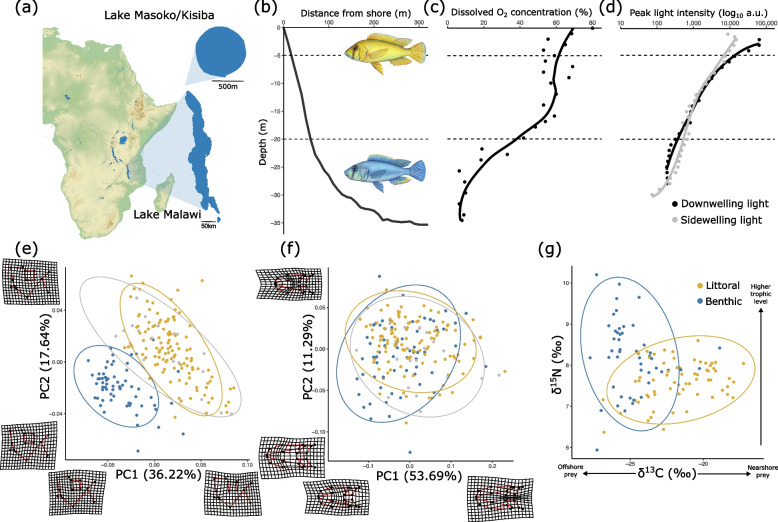
An overview of the Lake Masoko *Astatotilapia calliptera* system. **a** The location of Lake Masoko within Africa, relative to Lake Malawi. **b** Bathymetric profile of Lake Masoko (data from Turner et al. 2019 [37]; data collected in 2018). Included are illustrations of males of the two ecomorphs found in Lake Masoko: the yellow littoral ecomorph (above) that dominates the habitat < 5 m; and the blue benthic ecomorph (below) that dominates the habitat > 20 m. **c** Dissolved O_2_ concentration by depth, showing the oxycline at ~ 15 m (Delalande 2008 [38]; data collected 13/03/05) **d** The peak light intensity by depth for sidewelling (grey) and downwelling light (black) (data collection in summer 2018). **e** Principal Component Analysis (PCA) on the residuals of Procrustes coordinates on landmark data summarising the gross morphology of the lateral view of the head. Maximum and minimum morphological changes along each axis are illustrated as warped 2D meshes (*n* = 199). **f** PCA on the residuals of Procrustes coordinates from GPA on landmark data summarising the gross morphology of the ventral view of the head (*n* = 199). **g** Stable isotope analysis for ^13^C and ^15^N ratios from Lake Masoko cichlid muscle tissue (*n* = 113). Isotope data are from Malinsky et al. 2015 [31] and Carruthers et al. 2022 [33]

**Fig. 2:**
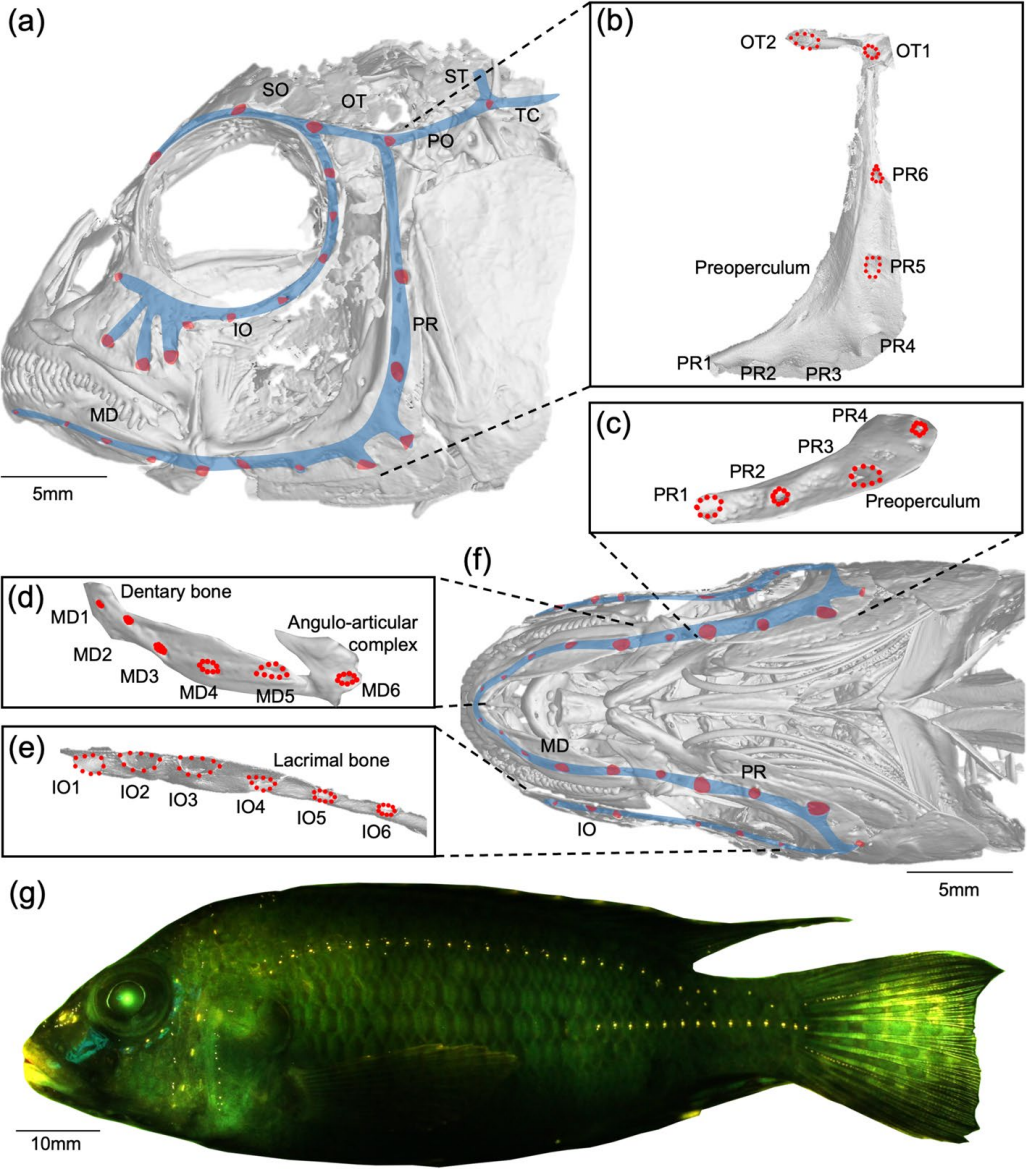
The cranial lateral line canal system of *Astatotilapia calliptera* from Salima, Lake Malawi – a sister population of the two *A. calliptera* ecomorphs found in Lake Masoko. **a** Annotated microCT scan of the lateral head, showing the cranial canal pores (red) and the approximate location of the cranial canals (blue). MD = mandibular canal; PR = preopercular canal; IO = infraorbital canal; SO = supraorbital canal; OT = otic canal; PO = post-otic canal; ST = supratemporal canal; TC = anterior trunk canal. **b**-**e** The landmarking regime of this study, used to approximate canal pore area. Each pore is uniquely coded. Red points are the landmark locations used; each pore is bounded by nine fixed landmarks placed equidistant along the outer edge of the pore. **b** The isolated preopercular and otic canals, and labelled pores. **c** The ventral-facing pores of the preopercular canal found within the preoperculum. **d** The isolated mandibular canal and pores, located within the dentary bone and angulo-articular bones. **e** The isolated infraorbital canal and pores, partially within the lacrimal bone. **f** Annotated microCT scan of the ventral view of the head. **g** A stitched photograph of a DASPEI stained *Astatotilapia calliptera* imaged under a fluorescent lamp and filter. Fluorescence images were also taken of the ventral view of the lower jaw, as the neuromasts there are not fully visible from the lateral perspective

Here we test for diversification in lateral line system morphology during the early stages of ecologically-associated speciation in a cichlid fish. We focus on Lake Masoko – also known as Lake Kisiba – a small crater lake in Tanzania which hosts two genetically divergent ‘ecomorphs’ of *Astatotilapia calliptera* [31] (Fig. 1a). These two populations have diverged within the last 1,000 years, following colonisation of the lake by riverine ancestors within the past 10,000 years [31, 32]. The ecomorphs exhibit distinct depth preferences and inhabit ecologically different environments (Fig. 1b-d). The “littoral” ecomorph dominates the shallow brightly-lit littoral habitat (< 10 m), whereas the “benthic” ecomorph dominates the deeper dimly-lit benthic habitat (> 20 m) (Fig. 1b, d). In addition, the two ecomorphs have distinct diets (Fig. 1g), male nuptial colouration (Fig. 1b), body shapes (Fig. 1e-f) and lower pharyngeal jaw morphology [31, 33, 34]. For the purpose of these analyses we define groups along a primary axis of genomic variation (PC1 of a genomic Principal Component Analysis), which correlates significantly with both phenotype and capture depth, categorising our subpopulations as “littoral”, “intermediate” and “benthic” (see “Materials and methods” section for more details). We also compare the lateral line morphology of these Lake Masoko subpopulations (Fig. 2) to that within the larger (700 + species) and older (1Ma) Lake Malawi radiation – of which *A. calliptera* is a member [35, 36] – enabling specific insight into whether patterns of early divergence we observe in Lake Masoko are reflective of those we see in the larger radiation.

## Results

### Cranial canal pore morphology

**Fig. 3:**
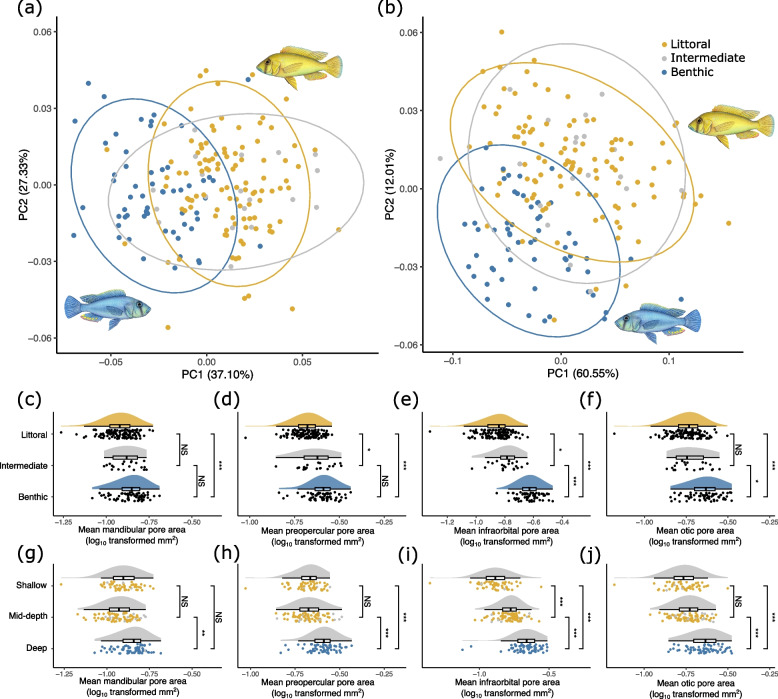
Divergence in the cranial canal lateral line system of *Astatotilapia calliptera* from Lake Masoko (*N* = 199). **a**-**b** Principal Components Analysis (PCA) on the residuals of generalised Procrustes analysis (GPA) on landmark data summarising cranial lateral line system pore morphology. Landmarks are grouped by **a** the lateral-facing pores of the otic and posterior preopercular canal; and **b** the ventral-facing pores of the mandibular, infraorbital and anterior preopercular canals. Also shown are 95% confidence ellipses for the three genomically-defined subpopulations. **c**-**j** Raincloud plots [39] of mean pore areas of the cranial lateral line canals of the subpopulations of *Astatotilapia calliptera* from Lake Masoko. All plots show partial residuals of the response variable after accounting for: standard length (log_10_ transformed); sex; gross lateral head morphology (PC1); and gross ventral head morphology (PC1). **c** Mean mandibular canal pore area grouped by genomic subpopulation. **d** Mean preopercular canal pore area grouped by genomic subpopulation. **e** Mean infraorbital canal pore area grouped by genomic subpopulation. **f** Mean otic canal pore area grouped by genomic subpopulation. **g** Mean mandibular canal pore area grouped by capture depth. **h** Mean preopercular canal pore area grouped by capture depth. **i** Mean infraorbital canal pore area grouped by capture depth. **j** Mean otic canal pore area grouped by capture depth. For all capture depth groupings, point colour indicates genetic subpopulation. For post-hoc comparisons (Tukey’s HSD): NS = not significant; *p < 0.05; **p < 0.01; ***p < 0.001

We found clear segregation of ecomorphs in Principal Component Analysis (PCA) morphospace, for both the combined lateral-facing (otic and preopercular canals, Fig. 3a) and ventral-facing pores (mandibular and infraorbital canals, Fig. 3b). Genomically intermediate fish exhibited significant overlap in their lateral line morphology with the littoral fish, with the benthic fish forming a semi-distinct cluster (Fig. 3a-b).

We estimated the area of each cranial canal pore within each specimen (as in Scott et al. 2023 [40]). We found significant differences between genetic subpopulations in the mean canal pore area in all four measured traits, with a consistent pattern of significantly larger cranial lateral line pore areas in the benthic fish than the littoral fish (Table 1; Fig. 3c-f; Table S7). Genetically intermediate fish (those with PC1 between the littoral and benthic ecomorphs in a genomic PCA, see “Materials and methods” section for details) exhibited significantly larger pore areas than the littoral fish within only the preopercular and infraorbital canal pores (Fig. 3d, e), while genetically benthic fish had significantly larger pore areas than intermediate fish in the infraorbital and otic canals (Fig. 3d-f). Results were largely similar when grouping individuals by capture depth (Table 2). In three of the four measured canals (preopercular, infraorbital and otic), fish caught in deeper waters (> 20 m) had significantly larger pores than those caught in the shallow water (< 5 m) (Table 2; Fig. 3h-j), whereas there was no significant difference in mandibular canal pore area (Table 2; Fig. 3g). Fish from the mid-depth zone (> 5 m & < 20 m) had significantly larger pores than those from shallow waters within the infraorbital canal (Fig. 3i).

**Table 1:** General linear models of mean pore area measurements for four cranial canals, testing for differences between genetically characterised subpopulations

Response	Predictors	Sum of squares	F	df	residual df	*P* value	Tukey contrasts	Post-hoc *p*-value
Mean mandibular pore area (log_10_)	Subpopulation	0.733	48.96	2	194	< 0.001^***^	L-B	0.008^**^
	Standard length (log_10_)	1.810	241.77	1	194	< 0.001^***^	I-B	1.000
	Sex	0.004	0.489	1	194	0.485	I-L	0.155
	Gross lateral head morphology (PC1)	0.0001	0.0001	1	194	0.995		
	Gross ventral head morphology (PC1)	0.0001	0.0015	1	194	0.969		
Mean preopercular pore area (log_10_)	Subpopulation	1.346	107.20	2	194	< 0.001^***^	L-B	< 0.001^***^
	Standard length (log_10_)	1.683	268.17	1	194	< 0.001^***^	I-B	0.303
	Sex	0.001	0.156	1	194	0.693	I-L	0.040^*^
	Gross lateral head morphology (PC1)	0.018	2.825	1	194	0.094		
	Gross ventral head morphology (PC1)	0.010	1.646	1	194	0.201		
Mean infraorbital pore area (log_10_)	Subpopulation	4.209	234.98	2	191	< 0.001^***^	L-B	< 0.001^***^
	Standard length (log_10_)	1.844	205.88	1	191	< 0.001^***^	I-B	< 0.001^***^
	Sex	0.000	0.039	1	191	0.844	I-L	0.021^*^
	Gross lateral head morphology (PC1)	0.019	2.12	1	194	0.148		
	Gross ventral head morphology (PC1)	0.027	3.07	1	194	0.082		
Mean otic pore area (log_10_)	Subpopulation	1.238	70.36	2	194	< 0.001^***^	L-B	< 0.001^***^
	Standard length (log_10_)	1.74	198.22	1	194	< 0.001^***^	I-B	0.013^*^
	Sex	0.007	0.757	1	194	0.375	I-L	0.848
	Gross lateral head morphology (PC1)	0.001	0.037	1	194	0.848		
	Gross ventral head morphology (PC1)	0.002	0.170	1	194	0.681		

Models are fit against a Gaussian distribution

*L* Littoral, *I* Intermediate, *B* Benthic

^***^p < 0.001; ^**^p < 0.01; ^*^ *p* < 0.05

**Table 2:** General linear models of mean pore area measurements for four cranial canals, testing for differences between fish from different capture depths

Response	Predictors	Sum of squares	F	df	residual df	*P*	Tukey contrasts	Post-hoc *p*-value
Mean mandibular pore area (log_10_)	Capture depth	0.805	53.63	2	194	< 0.001^***^	S-D	0.085
	Standard length (log_10_)	1.733	231.03	1	194	< 0.001^***^	M-D	< 0.004^**^
	Sex	0.007	0.89	1	194	0.341	M-S	0.442
	Gross lateral head morphology (PC1)	0.001	0.001	1	194	0.993		
	Gross ventral head morphology (PC1)	0.002	0.29	1	194	0.588		
Mean preopercular pore area (log_10_)	Capture depth	1.357	105.75	2	194	< 0.001^***^	S-D	< 0.001^***^
	Standard length (log_10_)	1.644	256.10	1	194	< 0.001^***^	M-D	< 0.001^***^
	Sex	0.001	0.156	1	194	0.694	M-S	1.000
	Gross lateral head morphology (PC1)	0.023	3.683	1	194	0.056		
	Gross ventral head morphology (PC1)	0.007	1.030	1	194	0.312		
Mean infraorbital pore area (log_10_)	Capture depth	3.934	176.35	2	194	< 0.001^***^	S-D	< 0.001^***^
	Standard length (log_10_)	1.812	162.46	1	194	< 0.001^***^	M-D	< 0.001^***^
	Sex	0.063	5.63	1	194	0.0186^*^	M-S	< 0.001^***^
	Gross lateral head morphology (PC1)	0.023	3.68	1	194	0.056		
	Gross ventral head morphology (PC1)	0.007	1.03	1	194	0.312		
Mean otic pore area (log_10_)	Capture depth	1.289	76.25	2	194	< 0.001^***^	S-D	< 0.001^***^
	Standard length (log_10_)	1.740	205.86	1	194	< 0.001^***^	M-D	< 0.001^***^
	Sex	0.028	3.25	1	194	0.073	M-S	0.149
	Gross lateral head morphology (PC1)	0.207	20.61	1	194	< 0.001^***^		
	Gross ventral head morphology (PC1)	0.300	2.98	1	194	0.086		

Models are fit against a Gaussian distribution

*S* Shallow, *M* Mid-depth, *D* Deep

^***^p < 0.001; ^**^p < 0.01; ^*^p < 0.05

We found no evidence of consistent variation in lateral line canal pore sizes between the sexes (Tables 1 and 2), and when all females were excluded from our dataset (31 of 191 fish) (Tables S1 and S3), a similar trend in pore size variation between ecomorphs persisted. We found that in almost all cases, pore area was entirely decoupled from gross head morphology, both from the lateral head and ventral head perspectives (Tables 1 and 2). A significant association was only observed for gross lateral head morphology, when grouping individuals by capture depth, and otic canal pore area was the response variable (Table 2).

### Superficial and canal neuromast counts

**Fig. 4:**
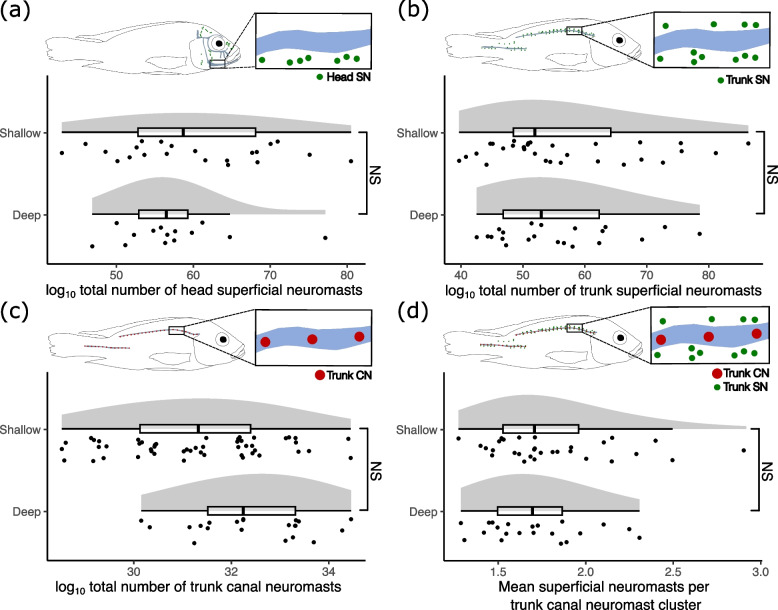
Neuromast counts for wild-caught *Astatotilapia calliptera* from Lake Masoko. Shallow individuals were caught above 5 m (*n* = 54), and deep individuals were caught below 20 m (*n* = 25). Comparisons are generalized linear models fit against a Poisson distribution, accounting for standard length (log_10_ transformed) and sex as covariates. **a** The total number of head superficial neuromasts. **b** The total number of trunk superficial neuromasts. **c** The total number of trunk canal neuromasts. **d** The average number of trunk superficial neuromasts per canal neuromast for both the anterior and posterior trunk canals. NS = not significant

We found no significant differences between shallow and deep-caught fish in the number of superficial neuromasts on the head (Fig. 4a), or the number of superficial neuromasts on the trunk (Fig. 4b, Table 3). There was no difference between ecomorphs in the number of trunk neuromasts within both branches of the trunk canal (Fig. 4c; Table 3). When comparing the average number of superficial neuromasts found clustered around each canal neuromast of the trunk canal (both anterior and posterior arms), we found no significant differences between the ecomorphs (Fig. 4d). In addition, we found no evidence for significant differences in neuromast number by standard length or sex (Table 3).

**Table 3:** Generalized linear models of neuromast counts for superficial neuromasts and canal neuromasts of the trunk, testing for differences between shallow (< 5 m) and deep caught (> 20 m) individuals. All neuromast count models are fit against a Poisson distribution

Response	Predictors	Estimate	Std. error	*P*-value
Total number of head superficial neuromasts	Capture depth	0.040	0.049	0.410
	Standard length (log_10_)	-0.265	0.306	0.387
	Sex	-0.116	0.089	0.194
Total number of trunk superficial neuromasts	Capture depth	0.012	0.038	0.760
	Standard length (log_10_)	-0.307	0.227	0.176
	Sex	0.052	0.054	0.330
Total number of trunk canal neuromasts	Capture depth	-0.038	0.046	0.417
	Standard length (log_10_)	0.057	0.263	0.828
	Sex	-0.009	0.069	0.890
Mean number of superficial neuromasts per trunk cluster	Capture depth	0.048	0.213	0.820
	Standard length (log_10_)	-0.359	1.271	0.777
	Sex	0.062	0.301	0.838

### Variation in Lake Malawi

**Fig. 5:**
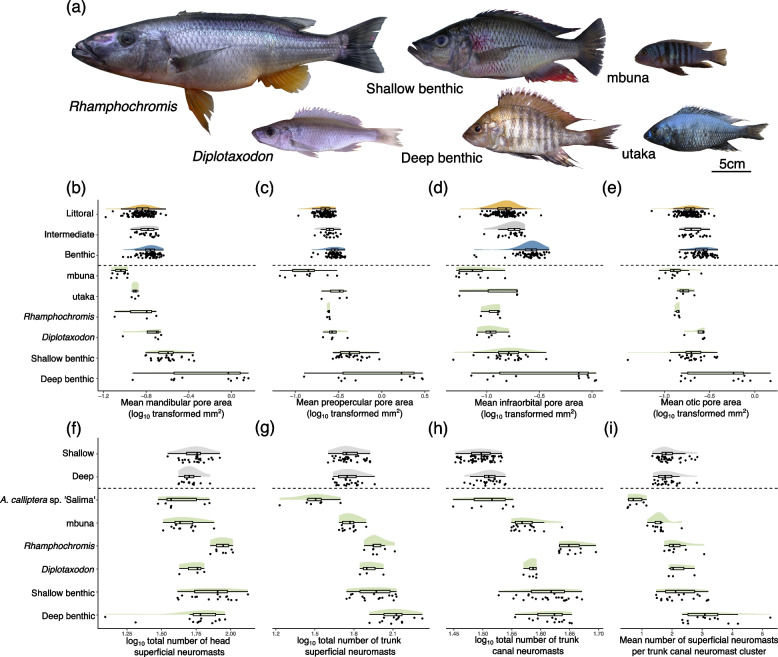
Comparison between the lateral line systems of the Lake Masoko *A. calliptera* ecomorphs and the broader Lake Malawi radiation. **a** Images of representatives of the six major clades of Lake Malawi haplochromine cichlids: *Rhamphochromis* (*Rhamphochromis woodi*); *Diplotaxodon* (*Diplotaxodon limnothrissa*); deep benthic (*Alticorpus geoffreyi*); shallow benthic (*Dimidiochromis strigatus*); mbuna (*Maylandia pulpican*); and utaka (*Copadichromis likomae*). **b**-**i** Comparisons of pore area and neuromast count measurements between Lake Masoko *Astatotilapia calliptera* (above dashed line) and the six main Lake Malawi cichlid lineages (green; below dashed line). Each point is an individual specimen, and all data are partial residuals from statistical models (Table S8). **b** Mean mandibular pore area. **c** Mean preopercular pore area. **d** Mean infraorbital pore area. **e** Mean otic pore area. **f** Total number of head superficial neuromasts. **g** Total number of trunk superficial neuromasts. **h** Total number of trunk canal neuromasts. **i** Mean number of superficial neuromasts per trunk canal neuromast cluster

Lineages with more pelagic lifestyles (*Rhamphochromis* and *Diplotaxodon*) had on average canals with narrow pore openings (Fig. 5b-d; Tables S1 and S5) and high numbers of neuromasts on the head and body (Fig. 5f-i; Table S6). When comparing the “shallow benthic” and “deep benthic” clades, both of which are characterised by associations with the substrate, the deeper-dwelling species on average exhibited larger canal pores, alongside a high number of superficial and canal neuromasts (Fig. 5e-h). The deep benthic clade, for example, consistently had significantly larger pores than the majority of other clades across all four cranial canals during post-hoc comparisons (Table S9). The rocky-shore dwelling mbuna, which are an immediate sister lineage to *A. calliptera* [36], had on average smaller cranial canal pores than many other clades, in particular than clades with associations with softer substrates (Table S9), exhibiting small pores and few neuromasts (Fig. 5).

Broadly, in terms of canal pore area, the degree of variation observed within Lake Masoko *A. calliptera* is of the same magnitude seen for interspecies comparisons of cichlids within Lake Malawi clades. In some cases, the extent of morphological disparity in Lake Masoko *A. calliptera* exceeds that observed between Lake Malawi clades. Those Lake Malawi clades with significantly different lateral line systems to Lake Masoko *A. calliptera* tend to inhabit niches or habitats which are absent from the smaller Lake Masoko. For example, the rocky-shore dwelling mbuna is on average characterised by smaller pores than most Lake Masoko subpopulations (Table S9), and *Rhamphochromis*, inhabiting the pelagic zone, has on average more neuromasts than Lake Masoko subpopulations.

## Discussion

In this study we investigated the evidence for divergence in components of lateral line system morphology between two recently separated populations [31]. We explored disparity in the cranial lateral line system by quantifying pore size in four of the cranial lateral line canals, using evidence from micro-CT scans. We also quantified divergence in the number of trunk canal and superficial neuromasts across the head and body using fluorescence imaging of stained neuromasts. After accounting for variation linked to body size and sex, we found the deep water benthic ecomorph had on average larger cranial lateral line canal pores than the shallow water littoral ecomorph (Fig. 3) (for example, the mean mandibular canal pore area was 0.152mm^2^ for the benthic and 0.116mm^2^ for the littoral ecomorph). However, we found no difference between the ecomorphs in number of canal or superficial neuromasts on the head or trunk (Fig. 4).

### Divergence in cranial canal morphology

Our observation of larger canal pores in the benthic ecomorph relative to the littoral ecomorph is suggestive of divergence in their sensory systems more broadly, and hence functional differences in their ability to detect hydrodynamic stimuli. The positioning of neuromasts in canals – and the widening of the pore openings to these canals – is thought to assist fishes that occupy dark or turbid conditions to detect highly variable current flows in their environments [7, 41–43], for example of the form generated by fish or zooplanktonic prey, or generated by hidden motile prey within muddy or sandy substrates [15]. There have been no measurements of turbidity in the Lake Masoko [37], which may vary by depth, but the intensity of light (Fig. 1d) and range of wavelengths of light both decline substantially by depth, and deeper water fish have experienced selection on their visual system in line with expectations from the measured light environment [31]. It is possible, therefore, that the different role that visual cues play in the deeper waters (in communication with conspecifics, behaviours such as schooling for predator avoidance, male-male competition for breeding territories, and male–female interactions during mating) may be accompanied by a modification in the role of lateral line-mediated detection of key signals [17, 44]. This may manifest as a reduction in the reliance on visual cues in the benthic ecomorph, compensated by an enhanced role for lateral line mechanoreception. However, given that there is evidence that vision is still key for communication at all depths within the lake [31], it is likely that any modifications to lateral line structures are not simply compensation for reduced reliance on vision.

The observed divergence in cranial canal morphology may be additionally linked to the differing diets of the two ecomorphs. Stable isotope analysis has revealed that the muscle of the littoral ecomorph is relatively enriched in ^13^C, reflective of a diet dominated by littoral arthropods, while muscle of the benthic ecomorph is relatively depleted in ^13^C and enriched in ^15^N, indicating a more offshore zooplantivorous diet and a higher trophic level [31, 33] (Fig. 1e). Previous research has identified that dietary grouping can be a useful predictor of cranial lateral line morphology across the Lake Malawi radiation [19].

We found no consistent association between pore size and trophic niche when comparing zooplankton feeders from Lake Malawi (i.e. within the “utaka” and *Diplotaxodon* clades) to species feeding on bottom-living invertebrates (i.e. within the “shallow benthic” and “deep benthic” clades) (Fig. 5), despite this being the case in Lake Masoko. Instead, within the Lake Malawi radiation, species with diets dominated by substrate-living invertebrate prey tend to have the largest pores [19] (Fig. 5). In particular, clades with substrate-associated or molluscivorous species, such as those of the “deep benthic” group, have significantly larger pores than most others (Table S9). Notably, however, there is pattern that where some species groups with similar diets living at different average depths are compared, those living in the deeper waters have larger cranial canal pores, as is the case for the “shallow benthic” vs. “deep benthic” groups) (Fig. 5b-e) (Table S9). This suggests that constraints imposed by both the dark light regime and the requirement to detect motile prey may combine to drive the evolutionary trajectory of lateral line system phenotypes.

### Evidence from canal and superficial neuromast counts

Similar to the cephalic canals, trunk lateral line canals are engaged with sensing alternating current flow, so may be best able to detect flow generated by proximate conspecifics or prey items [42]. By contrast, superficial neuromasts are thought to be primarily influenced by direct current, including abiotic water flow, thus informing behaviours such as rheotactic responses to background flow. However, superficial neuromasts are also thought to be utilised for sensing movement in low-background flow environments, particularly where visual cues may be limited. Many deep-sea fishes, for example, have an expanded number of superficial neuromasts on the head and body [43]. Similarly, the blind ecomorph of the Mexican tetra *Astyanax mexicanus* that inhabits cave environments where light and current are absent have vastly increased numbers of superficial neuromasts compared to sighted congenerics in surface habitats [22]. Thus, we may expect the benthic ecomorph of Lake Masoko *A. calliptera* to have more trunk canal neuromasts and more superficial neuromasts on their body, due to the deeper and darker environment they inhabit. However, we found no evidence of divergence between the deep and shallow living fish in the distribution of either neuromast type (Fig. 4b-c). This indicates that divergent light environments of Lake Masoko may not be driving sufficiently strong selection to lead to divergent adaptation of the trunk canal or superficial components.

Evidence suggests visual cues are of considerable importance for the benthic ecomorph in Lake Masoko, as evidenced by striking blue nuptial male colouration, and the shift in the visual spectrum towards higher wavelengths [31]. This is not the case for cave-living *A. mexicanus*, for which vision is entirely absent – in contrast to their sighted surface-living form [22]. This may explain the lack of difference between shallow and deep-living Lake Masoko fish in terms of neuromast number. It must also be acknowledged here that fish used for the neuromast dataset have not been genomically assigned to ecomorphs, and grouping individuals only by capture depth may be masking a significant association. There is gene flow between all three subpopulations in Lake Masoko [36], and capture depth does not necessarily correspond directly to genomic assignment of subpopulations.

In contrast with the lack of divergence observed in neuromast counts of Lake Masoko fish, we found substantial variation in neuromast number in the wider Lake Malawi radiation. Specifically, we observed that the number of superficial neuromasts was broadly associated with life history, encompassing diet and habitat (Fig. 5). The typically open-water feeding *Rhamphochromis* and *Diplotaxodon* clades, and the demersal feeding “shallow benthic” and “deep benthic” clades, had on average the greatest number of neuromasts in their trunk canals (Fig. 5f-i). Conversely, mbuna species inhabiting the rocky-shore littoral zone of Lake Malawi, that predominantly feed on epilithic algae and allied resources [26, 45] generally have few trunk canal and superficial neuromasts (Fig. 5f-i). Notably, *A. calliptera* had a similar number of neuromasts to the mbuna species. Although *A. calliptera* is typically omnivorous, it is also phylogenetically resolved as a sister species to the mbuna clade [36], perhaps indicative of a degree of phylogenetic constraint on lateral line phenotypes. *A. calliptera* from Lake Malawi had consistently fewer neuromasts than all subpopulations from Lake Masoko. This intraspecific variation may reflect some aspect of the differing ecologies of these fish, or another unknown aspect of life history. A larger sample size will be needed to fully resolve the evolutionary explanation for this disparity.

### Selection and constraints on lateral line system disparity

The evidence of divergence in lateral line phenotypes in Lake Masoko over a timescale of less than 1,000 years [31] (Fig. 3), combined with evidence of divergence across the broader Lake Malawi radiation that has evolved over the last one million years [36] (Fig. 5), is consistent with a role for natural selection in shaping lateral line phenotypes, and promoting broad-scale evolutionary divergence of these cichlid fishes. However, our results raise multiple issues that will require further investigation to address. Notably, our analyses have not confirmed a genetic basis for the observed cranial canal line variation in Lake Masoko *A. calliptera*. It is possible that observed phenotypic variation has arisen from developmental plastic responses to differing resource availability. Confirmation of a fixed genetic basis to this divergence will require quantification of the morphology of fish from each genetic background that have been reared in common garden conditions. Additionally, it may be possible to identify genetic variants associated with the trait in either hybrids [46] and/or by studying expression quantitative trait loci (eQTLs) [33]. Linking evidence from single nucleotide polymorphism data with expression data from wild Lake Masoko *A. calliptera* has proved successful for identification of key functional genes under selection that influence lower pharyngeal jaw shape, including documenting a role for genes associated with bone development [33, 34].

Our results show strong evidence for early divergence in aspects of the lateral line, though further evidence is required to conclusively identify the key drivers of divergence in the lateral line phenotypes of Lake Malawi haplochromines. Like visual systems, it is likely that multiple ecological factors mediate selection, including diet, habitat and social behaviours that can vary across ontogeny [47]. There are also likely to be phylogenetic constraints that limit standing genetic variation and therefore the phenotypes that selection can generate [19, 48]. Importantly, cephalic lateral line phenotypes are related to other key aspects of head morphology, including jaw, operculum and eye morphology [19, 49, 50]. Thus, there appear to be intrinsic constraints on the types of lateral line canal structures that can develop in the context of key aspects of morphology (Fig. 3a-b). It is possible, for example, that the relatively larger head of the deep-water benthic Lake Masoko ecomorph (Fig. 1b) [31] can reliably accommodate larger pores that would not be feasible in the shorter-jawed shallow water littoral ecomorph. Closer explorations of the covariance between lateral line canal structures and broader aspects of craniofacial morphology, both within and between ecomorphs, coupled with studies of lateral line system development [50–52], would help to resolve constraints and modularity of the system.

## Conclusions

There is now a wealth of evidence that ecological speciation in East African cichlids is enabled by adaptation to different habitats and trophic niches [23, 53], which in turn are facilitated by the evolution of divergent sensory systems – e.g. vision [1] – and ecomorphological traits – e.g. cichlid pharyngeal and oral jaws [5, 54]. In the Lake Masoko system, we are fortunate in being able to observe a potential incipient speciation event, where clear evidence of niche partitioning of populations accompanies lateral line system divergence, in addition to the more commonly studied divergent visual systems and ecomorphological phenotypes. Our results suggest the Lake Masoko system may provide opportunities to explore the evolution of the cranial lateral line canal system in the wild. Here, there is the potential for this mechanosensory system to be studied experimentally, to learn more about how regulation of gene expression changes during development and how lateral line morphology interacts with behaviour – for example during schooling [44, 55] and male-male competitive interactions [17]. We suggest that the lateral line system is a vital and intrinsic component of the functional morphology of fishes, which requires detailed consideration if we are to better understand mechanisms driving adaptive radiation in fishes.

## Materials and methods

### Cranial canal pore morphology – sample collection

For CT scanning, 191 individuals (160 males & 31 females) were caught at Lake Masoko using SCUBA in April 2018. Of these 48 were from the shallows (< 5 m), 83 were from the intermediate depth (mid-depth) (5-20 m), and 61 were from deep waters (> 20 m). We also included eight male individuals collected in August 2016, four caught shallow (< 5 m) and four caught deep (> 20 m), bringing our total dataset to 199 individuals. Our analyses were conducted i) on genetically-defined subpopulations, and ii) on fish grouped by collection depth. Genetic assignments to subpopulations were based on scores from a Principal Components Analysis (PCA) of SNPs derived from whole genome sequencing data of these same fish by Munby et al. 2021 [56]. Specifically, the PC1 scores from Munby et al. [56] show clear separation of ecomorphs, and thus define our genomic subpopulations. The ‘benthic’ individuals are defined as PC1 > 0.04 (*n* = 64), ‘littoral’ individuals are defined as PC1 < -0.02 (*n* = 115), and ‘intermediate’ individuals are defined as PC1 between -0.02 and 0.04 (*n* = 20). For all analyses and discussion herein, “littoral”, “intermediate” and “benthic” refer to genomically-defined subpopulations, whereas the terms “shallow”, “mid-depth” and “deep” all refer to capture-depth.

### Cranial canal pore morphology – CT scanning and data collection

Individuals selected for scanning were screened such that they were all of sufficient size and likely to be adults and did not exhibit any obvious morphological defects to render them unsuitable for scanning or further analysis. To visualise the cranial lateral line system anatomy, heads (all structures anterior of the posterior-limit of the operculum) were microCT scanned using a Nikon XTH225ST micro-computed tomography (micro-CT) system at the University of Bristol. Each scan covered two individuals and used 3141 projections. Voxel size for each scan was 20–30 µm. Scan resolution was determined by the size of the cranial lateral line canal pores, which were in turn determined by specimen size. Preliminary scans using similar specimens, and experience from previous scans [19] determined an appropriate resolution for subsequent scans. Image stacks were imported into VGStudio MAX 3.3.6 (Volume Graphics GmbH, 2016) and reconstructed into a 3D model. 2D images of the reconstructed model were captured from the ventral head perspective (showing the pores of the mandibular/infraorbital canals, and the lower arm of the preopercular canal), and the lateral head perspective on the left side (showing the pores of the lateral arm of the preopercular canal, and the otic canal) (Fig. 2a-b).

To summarise the morphology of the cranial canal lateral line system, we used tpsDig 2.31 [57] to draw curves of sliding semi-landmarks around the circumference of each pore, anchored by landmarks at its anterior limit. These curves were resampled, resulting in ten equidistant semi-landmarks for each pore. All landmarks were digitised by the same individual with only short breaks between landmarking sessions to minimise human error. We tested for human digitisation error by re-digitising 20 individuals’ cranial canal pores and testing for differences between our dataset and the re-digitised scans [58]. Analysis of variance revealed no significant difference between the two landmarking events (*F*_1,19_ = 0.512, *p* = 0.479), but significant differences between the 20 individual specimens (*F*_19,19_ = 9.011, *p* = 0.008) (Figure S1). After conversion from semi-landmarks to landmarks in tpsUtil32 [57], landmark coordinates were imported into R 4.2.1 [59] with the package geomorph 4.0.5 [60]. Image scale was accounted for when importing landmarks.

For the purpose of estimating pore area, the tenth landmark for each pore was discarded as its coordinates are the same as the first. The remaining nine landmarks formed the vertices of a polygon, of which the area was calculated using the package geometry 0.4.7 [61] in R 4.2.1 [59]. Resulting areas are approximations of the true pore area: although being a slight underestimate, we were consistent in our methodology across all specimens. We used this method to estimate the size of the five anterior-most pores of the mandibular canal (Fig. 2d), the six pores of the preopercular canal (Fig. 2b, c), the six pores within the infra-orbital canal (Fig. 2e), and two pores of the otic canal (Fig. 2b).

In addition to calculating the area of each pore, we used a landmark-based geometric morphometric approach to summarise the gross morphology of the head from both the lateral and ventral perspectives (Fig. 1e, f). Using images of reconstructed μCT scans, we developed a landmarking regime to summarise gross morphology (Figure S3) and repeated across all individuals. As with our lateral line data, coordinates of landmarks were imported into R 4.2.1 [59] in the geomorph package [60], accounting for variation in scale, rotation and translation.

### Neuromast imaging – sample collection

For the visualisation and imaging of superficial and canal neuromasts, 79 specimens of *Astatotilapia calliptera* (68 males & 11 females) were caught at Lake Masoko in October 2019 (Fig. 1a) using SCUBA. A total of 54 “shallow” individuals were caught at a target range of < 5 m, and 25 “deep” individuals were caught at > 20 m (Tables S2 and S4). Littoral fish were immediately transported to the Tanzania Fisheries Research Institute (TAFIRI) laboratory in Kyela, Tanzania in aerated barrels. Benthic fish were depressurised in holding barrels in the lake over two days before transportation. Fish were kept in holding tanks at TAFIRI Kyela before being processed.

### Neuromast imaging – fluorescence imaging and data collection

For imaging the neuromasts of these 75 fish, they were initially stained in 0.008% solution of the fluorescent dye DASPEI [2-(4-(dimethylamino)styryl)-N-ethylpridinium iodide; Fisher Scientific]. DASPEI is a vital mitochondrial dye, commonly used for the staining of epidermal mechanoreceptors and electroreceptors in teleosts and larval amphibians [62, 63]. It has been utilised to visualise both superficial and canal neuromasts in many fish groups, including cichlid fishes [12, 17, 40, 63]. After submersion in DASPEI solution, fish were subsequently euthanised using MS-222, an approved Schedule 1 method. Neuromasts of each fish were imaged on the left side of the body using a Canon EOS 500D DSLR camera and a Sigma 18–200 mm f/3.5–6.3 lens. Photos were taken in dark conditions with a Royal Blue lamp (465 nm) under a yellow glass longpass filter (500 nm) to remove interference. Images for each individual fish were stitched together in Fiji 1.51 [64] (Fig. 2g). Superficial and canal neuromasts were counted visually for each specimen where they a) were clearly visible; and b) exhibited variation between individuals. For all analyses “Total head superficial neuromasts” is defined as the sum of superficial neuromasts in the forehead, nose, pre-gill, post-gill and lower jaw regions (Figure S2a-b). “Total trunk superficial neuromasts” is defined as the sum of the superficial neuromasts associated with the anterior and posterior arms of the trunk canal (this includes any superficial neuromasts found in the trunk region but not associated with trunk canal neuromasts) (Figure S2a-b). “Total trunk canal neuromasts” is calculated as the sum of canal neuromasts in both the anterior and posterior trunk canals (Figure S2a-b). “Mean superficial neuromasts per trunk canal neuromast cluster” is related to the organisation of neuromasts in the cichlid disjunct trunk canal. Each trunk canal neuromast has clusters of superficial neuromasts around it, which were counted for each canal neuromast, then averaged across the entire length of the trunk canal (Figure S2b). This measure gives some indication of the density of superficial neuromast patterning across the fish trunk.

### Comparisons with Lake Malawi cichlids

Evidence from Lake Malawi cichlids shows patterns of cranial canal morphology can be clade-specific [19]. To expand on this, and to contextualise the extent of divergence in Lake Masoko, we calculated mean pore areas and counted neuromasts in representatives of the six major clades of Lake Malawi haplochromine cichlid radiation [36]. Specifically, we quantified pore areas from the four focal cranial canals (mandibular, preopercular, infraorbital and otic) in 52 species from Lake Malawi (*n* = 1 for each species), which were representative of the major ecomorphologically divergent clades in the species flock: the shallow rocky shore-dwelling mbuna; the shallow open water zooplanktivorous utaka; the deep-water predatory *Diplotaxodon*; the open water predatory *Rhamphochromis*; the shallow water benthic group; and the deep water benthic group [36] (Table S1). The microCT scans used for this analysis were from Edgley and Genner [19]. All microCT scanning, landmark digitisation and subsequent analysis were conducted as described above.

For neuromast comparisons we included several species representative of the diversity found in the major clades present in Lake Malawi [27, 36]. We imaged 12 different species within 6 groups (Tables S1 and S6). Individuals were sourced from or bred from existing stock in aquaria in the UK, at either Bangor University, the University of Hull or the University of Bristol. Between four and ten individuals of each of these were imaged in the same manner as Lake Masoko *A. calliptera* as outlined above, and neuromasts were counted using the same procedures as outlined above (Figure S2).

### Statistical analyses

To visualise the position of each fish in morphological space, we used a geometric morphometric approach for our gross morphology landmark data. We used a generalised Procrustes analysis (GPA) to align landmarks across specimens accounting for translation, orientation and scaling. We then conducted Principal Components Analyses (PCA) on Procrustes coordinates in geomorph 4.0.5 [60] for R 4.2.1 [59]. We repeated these analyses for both the lateral head (Fig. 1e) and ventral head (Fig. 1f) perspectives, to visualise the morphological variation among specimens. When accounting for gross head morphology in subsequent analyses we included PC1 of these PCAs as a covariate for general linear models (where pore area was the predictor variable). PC1 for both gross lateral and gross ventral head morphology were included in these cases. These PC1 variables summarise 36.22% and 56.39% of morphological variation respectively (Fig. 1e and f).

We repeated this analysis for our lateral line landmark data: using a generalised Procrustes analysis (GPA) to align landmarks across specimens, and a subsequent Principal Components Analyses (PCA) on the resulting Procrustes coordinates in geomorph 4.0.5 [60]. These analyses were conducted for the combined ventral-facing pores (mandibular canal, infraorbital canal and anterior preopercular canal – pores PR1-PR4) (Fig. 2c-f) and repeated for the combined lateral-facing pores (otic canal and posterior preopercular canal – pores PR5-PR6) (Fig. 2a-b).

For each of the four cranial canals (mandibular, preopercular, infraorbital and otic), we used log_10_ mean area of all pores as predictor variable in a general linear model (GLM), with: subpopulation (littoral, intermediate or benthic): sex; PC1 of gross lateral head morphology; PC1 of gross ventral head morphology; and log_10_ standard length as response variables (Table 1). Analysis was conducted in R 4.2.1, in the stats package [59]. We then used log_10_ mean pore area as a response variable in a GLM with capture depth (shallow < 5 m, mid-depth 5-20 m, deep > 20 m); sex; PC1 of gross lateral head morphology; PC1 of gross ventral head morphology; and log_10_ transformed standard length as response variables. For each GLM we used Tukey’s HSD (honestly significant difference) post-hoc tests and Bonferroni correction for multiple testing using R package multcomp 1.4–25 [65]. Plots of pore areas are partial residuals from these models, calculated in R packages emmeans 1.8.7 [66] and jtools 2.2.1 [67], visualised with sjplot 2.8.14 [68], showing differences according to subpopulation or depth of capture, while accounting for standard length and sex. We similarly tested for significant differences in neuromast counts between deep-caught and shallow-caught cichlids using generalized linear models with a Poisson distribution, including only capture depth (shallow < 5 m, deep > 20 m), standard length, and sex as predictor variables. Genomic data were not available for these neuromast-imaged specimens, and as such we only present here statistical comparisons of capture depth.

In order to visualise the extent of separation between subpopulations in a broader context, we constructed additional statistical models including representatives of the variation of the Lake Malawi adaptive radiation. For our CT scanning dataset, log_10_ mean area of pores was the response variable in a GLM with a gaussian distribution. Predictor variables for these models were “group” (Lake Masoko subpopulation or Lake Malawi clade), and the covariates log_10_ standard length; sex; gross lateral head morphology (PC1); and gross ventral head morphology (PC1). Gross lateral and ventral head morphology variables are from Edgley & Genner [19]. We conducted a Tukey’s honestly significant difference (HSD) post-hoc test with Bonferroni correction to compare groups (Table S9). For the neuromast count dataset, GLMs were constructed in a similar fashion, though using a poisson distribution (Table S8), and without the gross morphology covariables (Table S8). For the purpose of visualisation of comparisons, all data shown are partialized residuals from general linear models, accounting for differences according to sex and standard length (Fig. 5).

### Supplementary Information

**Supplementary Material 1.**

**Supplementary Material 2.**

## Data Availability

All code and raw data are available on github at the following link, and will be assigned a permanent DOI on acceptance: https://github.com/DuncanEdgley/Masoko_lateral_line_2023.git.
