# Supplementary material for: Lateral line system diversification during the early stages of ecological speciation in cichlid fish
Source: BMC Ecol Evol. 2024 Feb 20;24:24. doi: 10.1186/s12862-024-02214-5 (PMC10877828; doi:10.1186/s12862-024-02214-5)
Supplement: Supplementary file 2 — Supplementary Material 2. [file 12862_2024_2214_MOESM2_ESM.docx]

**SUPPLEMENTARY MATERIAL**

**
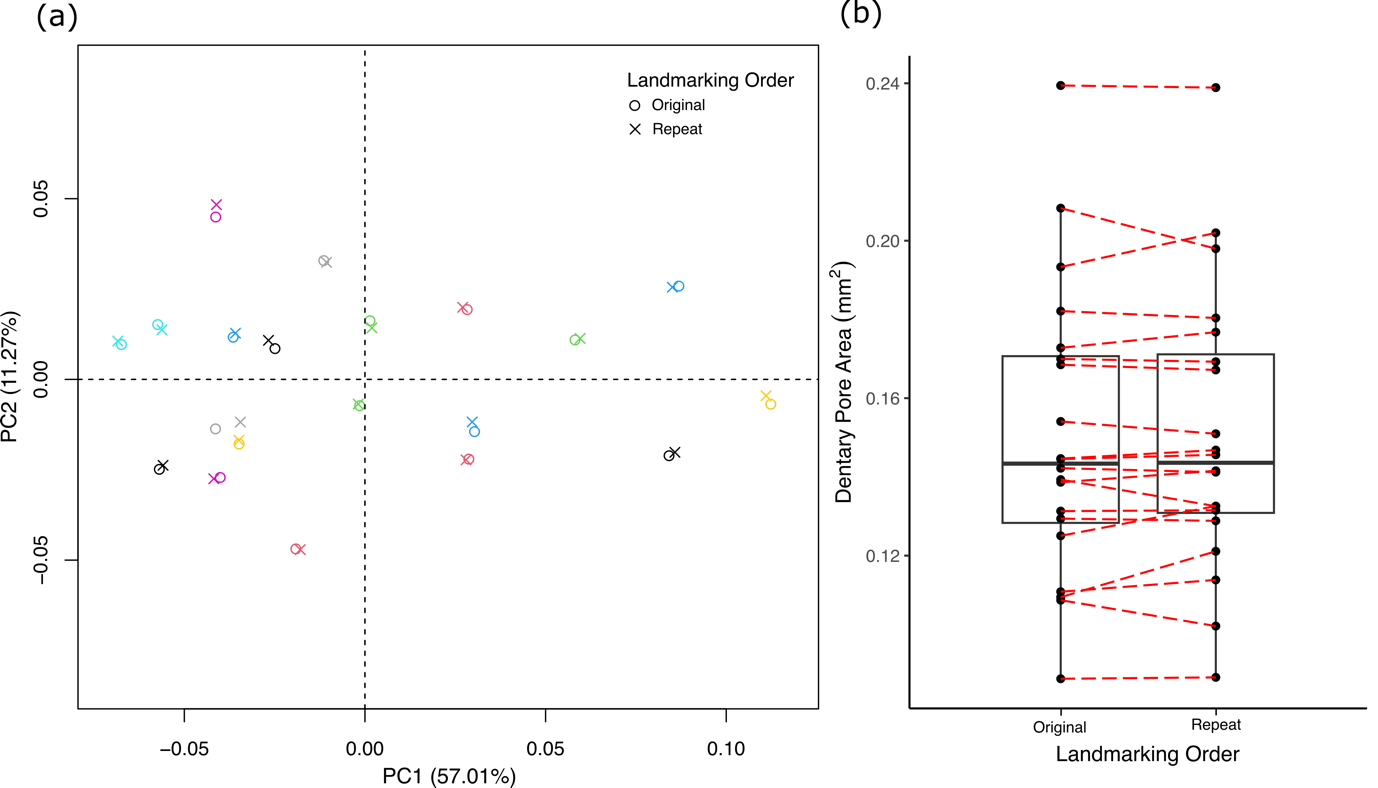
 Figure S1.** Calculation of digitisation error when phenotyping cranial canal lateral line anatomy. CT scans of 20 Lake Masoko *A. calliptera* were subjected to repeat landmarking events. Analysis of variance (ANOVA) showed that there was no significant difference between landmarking events (F_1,19_ = 0.0.512, p=0.47868), but highly significant differences between specimens (F_19,19_ = 9.011, p = 0.0079), indicating there is no evidence for human error significantly influencing our results. a) Principal Components Analysis (PCA) on Procrustes-transformed landmark coordinate data in these 20 Lake Masoko individuals. Point shape represents landmarking order (circle = original; cross = repeat), and each colour represents a different fish. b) Boxplot of the mean area of the mandibular canal pores (within the dentary bone) of 20 fish during original and repeat landmarking events.

**
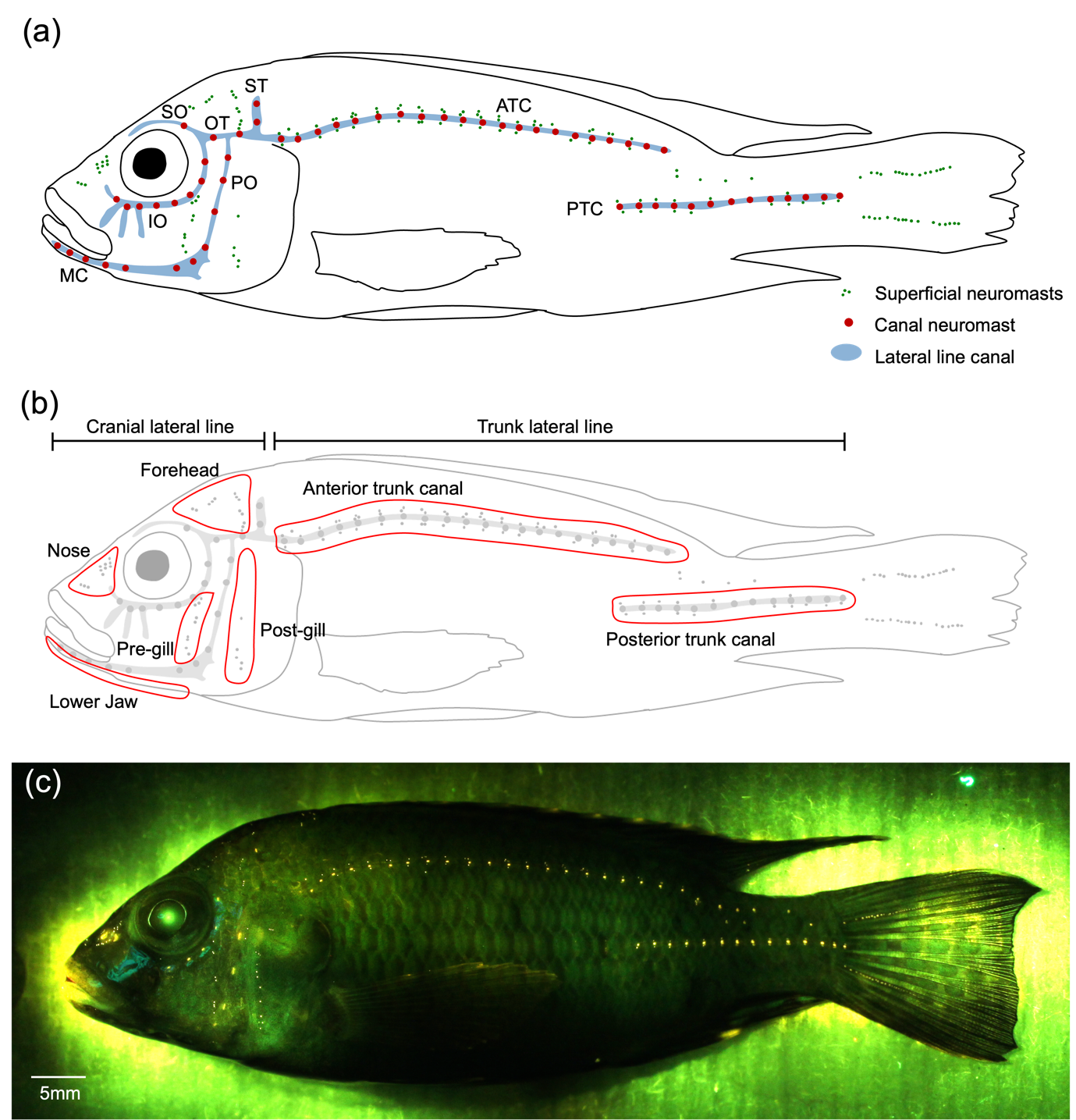
Figure S2.** Neuromast patterning of Astatotilapia calliptera from Salima, Lake Malawi. (a) Schematic overview of the lateral line system. Locations of superficial (green) and canal neuromasts (red) are shown, along with approximate location of lateral line canals (blue). MC = mandibular canal; IO = infraorbital canal; PO = preopercular canal; SO = supraotic canal; OT = otic canal; ST = supratemporal canal; ATC = anterior trunk canal; PTC = posterior trunk canal. (b) The neuromast counts used in this analysis. For both the anterior and posterior trunk canal, both superficial neuromasts and canal neuromasts were counted. For the cranial lateral line system, only superficial neuromasts were counted. (c) A stitched photograph of a DASPEI stained Astatotilapia calliptera.

**
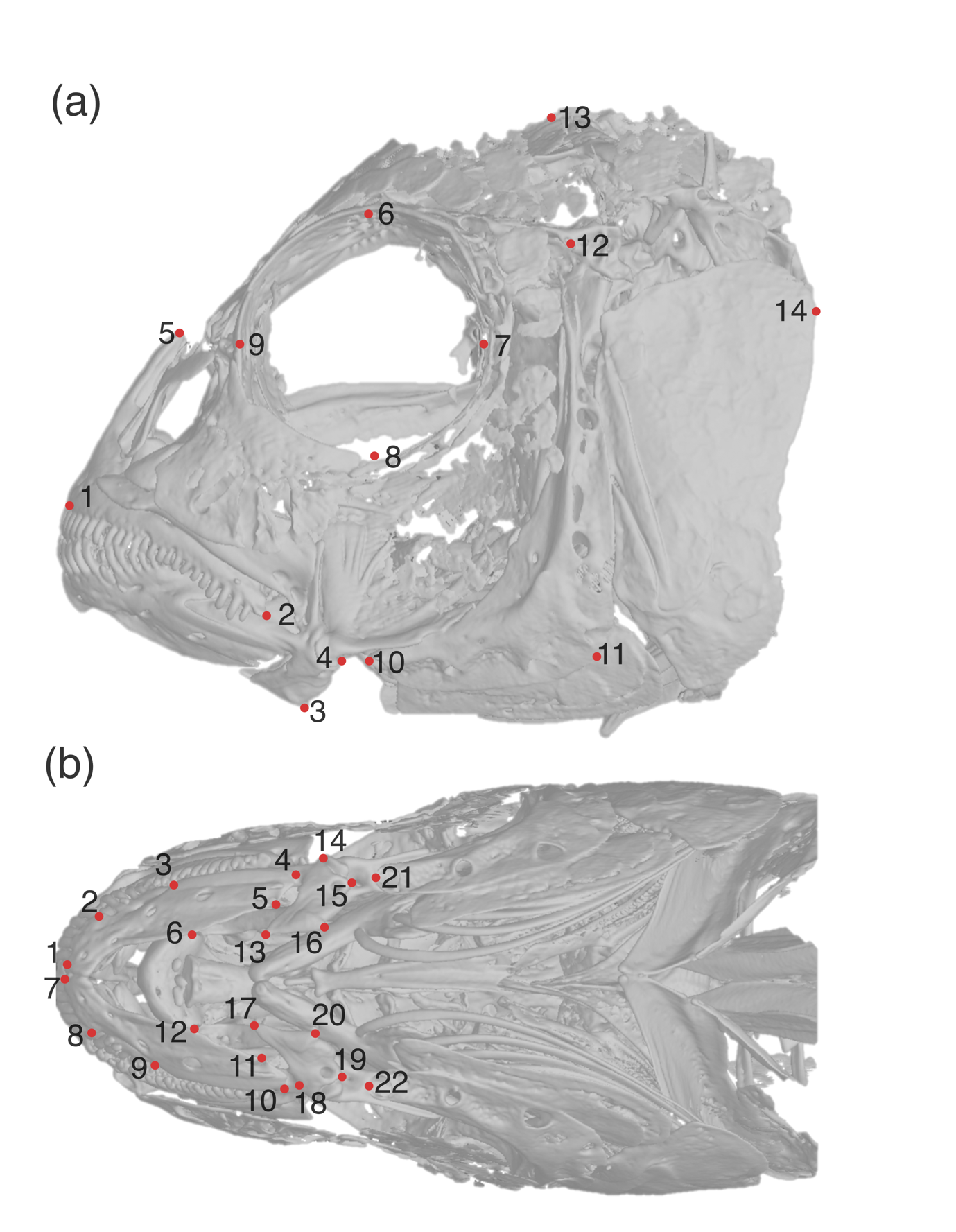
**

**Figure S3**. The landmarking regime used in this study, illustrated on a microCT scan of Astatotilapia calliptera from Salima, Lake Malawi. (a) The lateral view of the head and 14 associated landmarks digitised as follows: 1: anterior limit of maxilla; 2: posterior limit of oral cavity; 3: ventral limit of angulo-articular complex; 4: caudal limit of angulo-articular complex; 5: dorsal limit of premaxilla; 6: dorsal limit of bony orbit; 7: posterior limit of bony orbit; 8: ventral limit of bony orbit; 9: anterior limit of bony orbit; 10: anterior limit of preoperculum; 11: posterior limit of preoperculum; 12: dorsal limit of preoperculum; 13: tip of supraoccipital crest; 14: posterior limit of operculum. (b) The ventral view of the head and the 22 associated landmarks, digitised as follows: 1-6: landmarks around left mandibular bone at homologous points; 7-12: landmarks around right mandibular bone at homologous points; 13-16 landmarks around left angulo-articular complex at homologous points; 17-20 landmarks landmarks around right angulo-articular complex at homologous points; 21: anterior limit of left preopercular bone; 22: anterior limit of right preopercular bone.

**Table S1.** Summary table of species scanned in the CT dataset of this study. All individuals wild caught in either Lake Malawi or Lake Masoko. *Wild caught by S.M. Grant, location unknown. **Putatively undescribed species.

| **Group** | **Species** | **Sample size** | **Collection year** | **Sampling Location** |
| --- | --- | --- | --- | --- |
| Masoko Littoral | *Astatotilapia calliptera* (Masoko littoral) | 115 (84M, 31F) | 2018 | Lake Masoko |
| Masoko Intermediate | *Astatotilapia calliptera* (Masoko intermediate) | 20 (20M, 0F) | 2018 | Lake Masoko |
| Masoko Benthic | *Astatotilapia calliptera* (Masoko benthic) | 64 (64M, 0F) | 2018 | Lake Masoko |
| Malawi *A. calliptera* | *Astatotilapia calliptera* | 1 | 2011 | Lake Itamba, Tanzania |
| mbuna | *Cynotilapia zebroides* | 1 | 1997 | Monkey Bay, Malawi |
|  | *Genyochromis mento* | 1 | 1997 | Nkhata Bay, Malawi |
|  | *Labidochromis vellicans* | 1 | 2011 | Chiofu, Malawi |
|  | *Labeotropheus trewavasae* | 1 | 2012 | Unknown* |
|  | *Melanochromis loriae* | 1 | 1997 | Nkhata Bay, Malawi |
|  | *Maylandia aurora* | 1 | 2014 | Cape Maclear, Malawi |
|  | *Chindongo bellicosus* | 1 | 2014 | Cape Maclear, Malawi |
| utaka | *Copadichromis chrysonotus* | 1 | 2014 | Mangochi, Malawi |
|  | *Copadichromis likomae* | 1 | 2014 | Cape Maclear, Malawi |
|  | *Copadichromis sp*.’mloto yellow head black dorsal’ | 1 | 2014 | Cape Maclear, Malawi |
| *Rhamphochromis* | *Rhamphochromis esox* | 1 | 2004 | Nkhata Bay, Malawi |
|  | *Rhamphochromis* sp. ‘chilingali’ | 1 | 2004 | Lake Chilingali, Malawi |
|  | *Rhamphochromis* sp. ‘longiceps grey-back’ | 1 | 2005 | Dwangwa, Malawi |
| *Diplotaxodon* | *Diplotaxodon greenwoodi* | 1 | 2004 | Cape Maclear, Malawi |
|  | *Diplotaxodon limnothrissa* | 1 | 2004 | Cape Maclear, Malawi |
|  | *Diplotaxodon* sp. ‘macrops ngulube’ | 1 | 2005 | Nkhata Bay, Malawi |
|  | *Pallidochromis tokolosh* | 1 | 2005 | Kasuza, Malawi |
| Shallow Benthic | *Buccochromis aff. nototaenia* | 1 | 2014 | Lake Malombe, Malawi |
|  | *Ctenopharynx nitidus* | 1 | 2014 | Cape Maclear, Malawi |
|  | *Dimidiochromis compressiceps* | 1 | 2011 | Mangochi, Malawi |
|  | *Dimidiochromis dimidiatus* | 1 | 2014 | Cape Maclear, Malawi |
|  | *Dimidiochromis strigatus* | 1 | 2014 | Mangochi, Malawi |
|  | *Fossarochromis rostratus* | 1 | 1997 | Nkhata Bay, Malawi |
|  | *Protomelas kirkii* | 1 | 2014 | Mangochi, Malawi |
|  | *Hemitaeniochromis spilopterus* | 1 | 2005 | Monkey Bay, Malawi |
|  | *Hemitilapia oxyrhynchos* | 1 | 2014 | Mangochi, Malawi |
|  | *Lethrinops lethrinus* | 1 | 2014 | Lake Malombe, Malawi |
|  | *Lichnochromis acuticeps* | 1 | 2014 | Cape Maclear, Malawi |
|  | *Mylochromis anaphyrmus* | 1 | 2014 | Cape Maclear, Malawi |
|  | *Nimbochromis livingstonii* | 1 | 2012 | Unknown* |
|  | *Otopharynx tetrastigma* | 1 | 2014 | Cape Maclear, Malawi |
|  | *Placidochromis electra* | 1 | 2012 | Unknown* |
|  | *Placidochromis milomo* | 1 | 2005 | Monkey Bay, Malawi |
|  | *Sciaenochromis psammophilus* | 1 | 2004 | Maldeco Fisheries, Malawi |
|  | *Stigmatochromis modestus* | 1 | 2014 | Cape Maclear, Malawi |
|  | *Stigmatochromis macrorhynchus* | 1 | 1996 | Nkhata Bay, Malawi |
|  | *Taeniochromis holotaenia* | 1 | 2014 | Cape Maclear, Malawi |
|  | *Taeniolethrinops aff. praeorbitalis* | 1 | 2014 | Mangochi, Malawi |
|  | *Trematocranus placodon* | 1 | 2005 | Monkey Bay, Malawi |
|  | *Tyrannochromis nigriventer* | 1 | 1997 | Nkhata Bay, Malawi |
| Deep Benthic | *Alticorpus peterdaviesi* | 1 | 2005 | Monkey Bay, Malawi |
|  | *Aulonocara jacobfreibergi* | 1 | 2014 | Cape Maclear, Malawi |
|  | *Aulonocara aff. nyassae* | 1 | 2014 | Cape Maclear, Malawi |
|  | *Aulonocara* sp. ‘copper’ | 1 | 2005 | Monkey Bay, Malawi |
|  | *Aulonocara* sp. ‘yellow collar’ | 1 | 2014 | Cape Maclear, Malawi |
|  | *Aulonocara stuartgranti* | 1 | 2012 | Unknown* |
|  | *Lethrinops gossei* | 1 | 2005 | Tukombo, Malawi |
|  | *Lethrinops* sp. ‘oliveri’ | 1 | 2014 | Cape Maclear, Malawi |
|  | *Lethrinops* sp. ‘zebra’ | 1 | 2005 | Nkhata Bay, Malawi |
|  | *Placidochromis platyrhynchos* | 1 | 2014 | Cape Maclear, Malawi |
|  | *Placidochromis polli* | 1 | 2005 | Monkey Bay, Malawi |

**Table S2.** Summary table of neuromast imaging specimens used in this study.

| **Group** | **Species** | **Sample size** | **Sourced from** | **Imaging location** |
| --- | --- | --- | --- | --- |
| Shallow | *Astatotilapia calliptera* (Masoko Shallow) | 54 (46M, 8F) | Lake Masoko (wild) | Kyela, Tanzania |
| Deep benthic | *Astatotilapia calliptera* (Masoko Deep) | 25 (22M, 3F) | Lake Masoko (wild) | Kyela, Tanzania |
| *A. calliptera* "Salima" | *Astatotilapia calliptera* (Salima) | 6 | Lake Malawi (aquarium) | Bangor University |
| mbuna | *Maylandia zebra* | 6 | Lake Malawi (aquarium) | Bangor University |
|  | *Maylandia estherae* | 5 | Lake Malawi (aquarium) | Hull University |
|  | *Labeotropheus trewavasae* | 5 | Lake Malawi (aquarium) | Hull University |
| *Rhamphochromis* | *Rhamphochromis* sp. “chilingali” | 5 | Lake Malawi (aquarium) | Bangor University |
|  | *Rhamphochromis longiceps* | 5 | Lake Malawi (aquarium) | Hull University |
| *Diplotaxodon* | *Diplotaxodon limnothrissa* | 5 | Lake Malawi (aquarium) | Hull University |
| Shallow benthic | *Nimbochromis livingstonii* | 5 | Lake Malawi (aquarium) | Hull University |
|  | *Protomelas* sp. "johnstoni solo" | 5 | Lake Malawi (aquarium) | Hull University |
|  | *Otopharynx lithobates*  *Nyassochromis microcephalus* | 10  5 | Lake Malawi (aquarium)  Lake Malawi (aquarium) | Bristol University  Hull University |
| Deep benthic | *Aulonocara stuartgranti* | 13 | Lake Malawi (aquarium) | Bangor University |
